# Association between umbilical cord hygiene and neonatal sepsis among neonates presenting to a primary care facility in Nairobi County, Kenya: a case-control study

**DOI:** 10.12688/f1000research.19544.2

**Published:** 2019-07-30

**Authors:** Phoebe K. Moraa, Marshal M. Mweu, Peter K. Njoroge

**Affiliations:** 1 School of Public Health, College of Health Sciences, University of Nairobi, Nairobi, Kenya

**Keywords:** Neonatal sepsis, Umbilical cord hygiene, Cord care practices, Case-control study, Primary care setting

## Abstract

**Background: **Three-quarters of all annual neonatal deaths in developing countries are attributable to neonatal sepsis. In primary care settings, poor cord hygiene due to improper handling of the infant’s cord is a major contributor to the occurrence of neonatal sepsis. The objective of this study was to describe the umbilical cord practices among mothers attending a primary care facility, assess the relationship between umbilical cord hygiene and neonatal sepsis, its impact on the population, as well as the influence of other neonatal and maternal factors on this relationship.

**Methods: **A case-control study was conducted to assess the umbilical cord hygiene-neonatal sepsis relationship among neonates attending a primary care facility between August and October 2018. All cases were selected, while controls were systematically random sampled, as per study eligibility criteria. Exposure variables were summarized using descriptive statistics. A multivariable logistic regression model was fitted to evaluate the association between umbilical cord hygiene and neonatal sepsis adjusting for the effect of potential confounders. Subsequently, a population attributable fraction (PAF) was estimated.

**Results: **The proportion of mothers with improper hygiene was 35.3%: 72.1% among the cases and 16.3% among the controls’ caregivers. The odds of neonatal sepsis were 13 times higher (OR=13.24; 95% CI: [7.5; 23.4]) among infants whose caregivers had improper hygiene compared to those who had proper hygiene. None of the neonatal and maternal covariates confounded the umbilical cord hygiene-neonatal sepsis association. This odds ratio gave a PAF of 66.7% (95% CI: 62.5; 69.0).

**Conclusions: **Improper cord hygiene is prevalent in this low resource setting. Improper cord hygiene has a strong positive association with neonatal sepsis. Observing good cord care practices could avert up to 67% of newborn infections. This calls for inclusion of comprehensive cord care practices in the antenatal care educational package.

## Introduction

Worldwide, neonatal mortality (death occurring within the first 28 days of life) accounted for 45.1% of all child deaths in 2015, representing a 15% increase over a span of 15 years ^1^. The leading causes of neonatal mortality globally are preterm birth complications, intrapartum-related events and neonatal sepsis ^1, 2^. These three constitute 75% of all neonatal deaths ^3, 4^. In the developing world, septicemia accounts for 1.6 million neonatal deaths per year ^2, 5^ and around 10-30% of neonatal deaths in Kenya ^6^.

Owing to the non-specificity of neonatal sepsis’ presentation in neonates, there has been a general lack of consensus on the definition of neonatal sepsis ^7^. Nevertheless, Shane *et al*. ^8^ define neonatal sepsis as a bacterial, fungal or viral systemic condition characterized by bio-physiological changes (e.g. abnormal leucocyte count, aberrant temperature or even tachycardia), clinical symptoms (e.g. presence of fever, feeding difficulties or umbilical discharge) and attended by significant morbidity and mortality ^8^.

Although maternal and neonatal factors are important risk factors for neonatal sepsis, umbilical cord hygiene represents a key determinant ^9– 11^. A hygienic umbilical cord refers to a dry umbilical stump without signs of redness, warmth, swelling, pain, foul smell or pus ^12, 13^. To maintain a hygienic cord, proper umbilical care is necessary. Appropriate care could be achieved by either applying methylated spirit/chlorhexidine to the base of the cord, air drying the cord to allow for natural healing or sponge-bathing neonates without immersing them in water ^14, 15^. The World Health Organization (WHO) recommends that dry cord care be employed within health facilities or home deliveries taking place in low mortality settings (less than 30 deaths per 1000 births). Chlorhexidine is advocated for home births within high neonatal mortality settings, particularly, as a substitute for harmful traditional compounds ^16^.

The probability for entry of pathogenic micro-organisms through the umbilical cord is high in low-resource settings ^17, 18^. This could be attributable to the prevailing sub-optimal hygienic conditions in the environment of the baby that could result in a localized umbilical cord infection (omphalitis) ^19^, with potential spread of the microorganisms into the bloodstream via the patent umbilical vessels resulting in septicemia or infection of other organs ^20^. Although clean birth practices are highly advocated for because of their role in averting the risk of omphalitis and neonatal infection, in many developing settings, cultural norms that dictate cord care practices may compromise cord hygiene ^11, 21^. In Kenya, as in other developing settings, the rationale for applying a wide variety of substances on the cord is to hasten cord separation and healing ^14, 17, 22, 23^. These substances, which include cow dung, charcoal, hot fermentation, mustard oil, ghee, ash or other non-septic applications, are significantly correlated with an increased risk of omphalitis and neonatal sepsis ^9, 10, 24^.

In Kenya, despite a neonatal mortality rate of 22 deaths per 1000 births ^6^, available guidelines on cord care are sketchy – with a sole focus on substance application, i.e. use of 4% chlorhexidine ^12^. This deficiency may predispose mothers to suboptimal cord care practices that could lead to omphalitis and thus neonatal sepsis. Despite the importance of proper cord care in the prevention of neonatal infection, review of published literature reveals a dearth of studies that demonstrate the association between umbilical cord hygiene and systemic infection especially in poor settings ^15, 24, 25^; with a sizeable number of studies paying attention to other factors associated with neonatal sepsis ^9, 25– 27^.

The objective of this study was to describe the umbilical cord practices among mothers attending a primary health care facility, assess the relationship between umbilical cord hygiene and neonatal sepsis, its impact on the population, as well as the influence of other neonatal and maternal factors on this relationship. Given the insufficient guidelines on cord care practices in Kenya, a critical understanding of the significance of good cord care on prevention of neonatal sepsis is central to informing decisions aimed at strengthening national guidelines on appropriate cord care practices as part of primary prevention strategies.

## Methods

### Study design and setting

A facility-based case-control study design was employed to identify the determinants of neonatal sepsis. The rationale for the choice of the design relates to the rarity of neonatal sepsis within the facility’s neonatal catchment population, thus rendering the health centre a ready source of case patients. Although population-based controls would conceivably be more preferable, potential differences in health-seeking behavior between hospital and population-sourced controls suggested the need to recruit controls from the same facility as cases. The study conformed to the STROBE guidelines for reporting of a case-control study ^28^.

The study was conducted at the Kahawa Health Centre (KHC) which is a level three state-run facility in the northern part of Nairobi County. The estimated catchment population for this health centre is about 52,193 persons and includes the adjacent peri-urban localities. Most of these areas are predominantly informal settlements characterized by overcrowding, with 99% of inhabitants being young adults. Anecdotal reports connote high neonatal mortality rates in this area.

### Study population and eligibility of participants

The study population comprised all neonates presenting to KHC for pediatric services during the span of August–October 2018. Case and control patients were selected from this population based on a predefined set of eligibility criteria. All primary visit neonates (incident cases) and infants whose guardians had consented to participation were included. Premature babies with gestational age less than 37 weeks, babies who had a lower than 2000 g birth weight and neonates with congenital anomalies were excluded from the study.

### Case definition and recruitment

A case patient was a 0-28 day-old neonate, a resident of the study area, presenting to KHC during the study period with an elevated axillary temperature of ≥37.5°C and any one of the following symptoms of infection: purulent discharge (from ear/eye/umbilicus), respiratory distress (cyanosis, grunting, nasal flaring and chest wall indrawing)/fast breathing (more than 60 breathes/minute), severe abdominal distension, poor difficulty feeding (persistent vomiting (last three feeds)/refusal to feed/inability to suck/weak suck), altered mentation (lethargic/unconsciousness/convulsions) or skin changes (deep jaundice/ periumbilical redness) ^29^. Considering that KHC registers around two to three neonatal sepsis cases per day, to attain the computed sample, all cases (who met the aforementioned eligibility criteria) presenting to the facility within the study period were prospectively recruited. Recruitment of cases occurred at pediatric outpatient consultation rooms.

### Control definition and recruitment

Controls were neonates similarly defined as cases (though devoid of sepsis symptoms), presenting to the well-baby clinic during the same two-month time period. Controls were systematically random sampled from the well-baby clinic of the facility frequency-matched to the cases by the day of presentation.

### Primary exposure definition

A definition for umbilical cord hygiene was adopted from WHO’s “five cleans” for postnatal care of the stump ^20^, based on indicators that comprised: the method of folding the napkin, rooming-in, bathing, handwashing and substance application practices, as reported by caregivers. An aggregate score equal to or above the median would constitute good cord hygiene, while scores below the median would be considered as poor hygiene.

### Sample size determination

As specified by Kelsey *et al*. ^30^ for case-control studies, the required sample size was derived:

$$n_{1}=\frac{{\left(Z_{\alpha }+Z_{\beta }\right)}^{2}\bar{p}\bar{q}\text{(}r+\text{1)}}{r{\text{(}p_{\text{1}}-p_{2}\text{)}}^{\text{2}}}$$

$$p_{1}=\frac{p_{\text{2}}OR}{1+p_{2}(\text{O}R-1)}$$

$$n_{2}=rn_{1}$$

$$\bar{p}=\frac{p_{1}+rp_{2}}{r+\text{1}}$$

$$\bar{q}=1-\bar{p}$$

Where: *n*_1_ = the number of cases; *n*_2_ = the number of controls; *p*_1_ = the proportion of cases with an unhygienic umbilical cord; *p*_2_= proportion of controls with an unhygienic umbilical cord specified at 37.6% based on a previous study ^31^. Notably, Z _α_ = 1.96 for the 2-tailed confidence level of 95%; Z _*β*_ = −0.84 for the desired statistical power of the study set at 80%; and *r*=2 as the specified ratio of controls to cases to enhance the study power. The odds ratio (OR) for the umbilical cord hygiene-neonatal sepsis association was estimated at 2. With an anticipated 5% non-response rate, the required sample size was 312: 104 cases and 208 controls.

### Study variables

**Figure 1.**
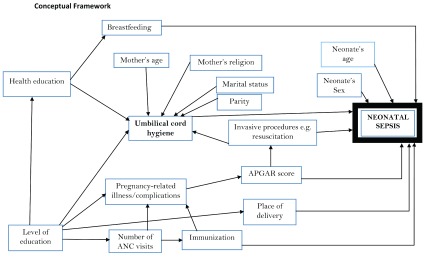
Causal diagram of umbilical cord hygiene and other factors thought to influence on neonatal sepsis occurrence among neonates at Kahawa Health Centre, Kenya.

Other than the primary exposure variable, the other predictor variables were maternal and neonatal factors. These were gathered using a semi-structured questionnaire, available as *Extended data* ^32^. Maternal factors consisted of socio-demographic factors (age of mother, level of education, marital status, parity and religion), the antenatal history of the mother (number of antenatal care (ANC) visits, history of receiving health education, tetanus toxoid immunization, prenatal maternal bacterial infection, birth attendance, place of delivery and type of delivery) and post-natal history factors (history of illness or pregnancy related complications such as postpartum depression, nutritional status or other comorbidities). Neonatal risk factors included low APGAR scores of <7 at 5 minutes (whose signs included scores of appearance, pulse, grimace, activity, and respiration), neonate’s age, sex and invasive procedures (use of medically invasive instruments/resuscitation at birth). Table 1 displays assessment of these variables. Figure 1 provides a conceptual framework of the relationship between the aforementioned predictors and the outcome.

**Table 1.** Independent variables together with their measurements.

Variable	Measurement of variable
Age of mother (continuous)	Expressed in years.
Mother’s level of education (ordinal)	The level of education attained by the mother. Classified into four levels: 1=No formal education, 2=Primary school, 3=High school education or 4=College/graduate education.
Marital status (nominal)	Captured in three categories: Single, Married or Others (divorced, widowed and separated).
Mother’s religion (nominal)	Expressed as Protestant, Catholic, Orthodox, Muslim or Pagan.
Place of delivery (nominal)	Grouped into two levels: Health institution or home delivery.
Type of delivery (nominal)	Mothers delivery categorized into three classes: Cesarean section (CS), Spontaneous vaginal delivery (SVD) or Instrumental (forceps/vacuum).
Health education (nominal)	Mothers were ranked by whether they had received antenatal education on cord care or not received.
Number of ANC visits (ordinal)	The number of ANC visits made by the mother. Captured as 0,1, 2, 3 or ≥4.
Immunization (nominal)	Tetanus toxoid-containing vaccines are administered at recommended intervals in pregnant women. The rationale for this vaccine is to protect both the mother and her child from tetanus during delivery ^33^. Mothers were classified into two groups: Immunized and Not immunized.
Pregnancy-related complications (nominal)	This was inclusive of all labor-related complications such as, premature rupture of membranes (PROM), chorioamnionitis/meconium aspiration syndrome (MAS) and elevated maternal temperature. It also included any history of bacterial infection in pregnancy. Measured in two categories: Present and Not present.
Neonate’s age (continuous)	Captured in days.
APGAR score (discrete)	APGAR (Appearance, Pulse, Grimace, Activity, and Respiration) scores are assigned to the newborn at 1, 5 and 10 minutes from the moment of birth. The signs observed and scored include heart rate, respiration, muscle tone, reflex irritability and color ^34^. The 5-minute score has been correlated with developmental vulnerability ^35^. Actual values of APGAR score at five minutes were recorded from the Mother and Child Health Booklet Kenya.
Invasive procedures e.g. resuscitation, ventilator support, intravenous line (nominal)	Resuscitation at birth using biomedical techniques. Retrieved from the Mother and Child Health Booklet Kenya. Represented by two categories: Done and Not done.
Parity (discrete)	Measured as number of children a mother had.
Neonates’ Sex (nominal)	The neonate’s sex captured as either male or female.
Cord exposure (nominal)	Mothers were questioned if cord was kept exposed, that is, if napkin was folded below or above the stump. Two groups were generated: Above the cord and Below the cord.
Substance application (ordinal)	Scaled into four levels: 0=Saliva/Ash, 1=None (air-drying)/Water, 2=Silver sulphadiazine/Topical antibiotic, 3=Surgical spirit/Chlorhexidine. In regards to treatment efficacy in reduction of cord infection methylated spirit and 4% chlorhexidine are comparable ^36, 37^. Effectiveness of chlorhexidine at cord healing is better than either silver sulphadiazine, topical antibiotic, povidone iodine or dry cord care ^38, 39^. Further, there is no significant difference between silver sulphadiazine, povidone iodine or topical antibiotic such as bacitracin ^39^. Equally, use of dry cord care was found to be commensurable to cleaning with water ^39^. Application of saliva or other traditional substances has been shown to predispose to omphalitis as compared to air drying ^40^.
Hand washing (ordinal)	Handwashing graded into two categories: 0=No, 1=Yes. Further the substance used to wash hands was categorized into three levels: 0=None, 1=Water only and 2=Water and soap.
Breastfeeding practice (ordinal)	Mothers were asked if early breastfeeding was initiated and if exclusivity of breastfeeding was practiced. Mother-neonate pair were in three sets: 0=Within one hour, 1=One-six hours, 2=More than six hours. For exclusivity, three groups were generated: 0=Breastmilk, 1=Formula, 2=Mixed, 3=Other.
Bathing method (nominal)	Neonates were bathed in either of two ways: Immersion bathing or sponge bathing.

### Ethical considerations

The research commenced after receiving written clearance from the Kenyatta National Hospital (KNH)-University of Nairobi (UoN) Ethics and Research Committee (P438/06/2018) and the Nairobi County Health Services (Ref. No. CMO/NRB/OPR/VOL.1/2018/91). Additionally, written informed consent was obtained from the mother/index care-giver for their neonate’s participation in the study.

### Minimization of biases

Prior to commencing the data collection, two research assistants were trained on screening patients, complete neonatal medical examination and standardized interview techniques to reduce interviewer bias. Additionally, caregivers could have resorted to unhygienic cord practices (such as harmful applications) as a way to treat an already septic neonate. Hence, the possibility of reverse causality was reduced by focusing on incident cases. Attempts to minimize recall bias were made by referencing the mother-child booklet to ascertain information regarding some antenatal and perinatal information such as the number of antenatal visits, the neonate’s date of birth, neonatal APGAR score and resuscitation history.

### Statistical analysis

The questionnaires were checked for completeness and qualitative data coded. The data were double-entered by two independent data entry clerks into EpiData version 3.1 spreadsheet. The principal researcher cross-checked the computerized data base against the questionnaires that had been administered. The dataset was exported to Stata software, version 13 (Stata Corporation, College Station, Texas, USA) for cleaning and analysis. For continuous variables’ descriptive statistics, data were summarized by means, medians and ranges. For categorical variables, data were summarized using frequency tables, proportions and percentages.

Scoring of the umbilical cord hygiene variable’s five components was standardized such that those responses that were desirable as per WHO essential newborn care guidelines received a higher value (≥1). A value of zero was awarded to responses inconsistent with these guidelines ^41– 43^. A total score was then reached by summing up the individual component’s scores. Notably, a cord with an aggregate score below the median score of 7 was designated as improper cord hygiene, whereas one with a score equal to or above the median was deemed to have proper cord hygiene.

A logistic regression model was used to assess the crude association between umbilical cord hygiene and neonatal sepsis. For a sensible interpretation of APGAR score’s effect on neonatal sepsis, it was grouped into two categories ^27^: ≥7 or <7. To evaluate the potential confounding effect of neonatal and maternal factors on the umbilical cord hygiene-sepsis relationship, each of the predictors was screened for unconditional associations with neonatal sepsis at a 5% significance level. Qualifying variables were further screened for a significant association with umbilical cord hygiene at similar level of significance.

Variables that met these criteria were considered as potential confounders to the cord hygiene-neonatal sepsis relationship and therefore were included in a multivariable model to adjust for their confounding effect on this relationship. Here, a backward step-wise approach was applied to eliminate variables if there was not more than a 30% change in the regression coefficient for umbilical cord hygiene upon their exclusion ^44^. To evaluate the impact of umbilical cord hygiene in the neonatal population (the proportion of neonatal sepsis that could be prevented by adhering to proper umbilical cord care), a PAF was computed as described by Dohoo *et al*. ^44^

$$PAF=pd\;\left(\frac{aOR-1}{aOR}\right)$$

Where: *PAF* is the population attributable fraction; *pd* is the proportion of total cases in the population arising from improper cord hygiene; *aOR* is the adjusted odds ratio for cord hygiene derived from the multivariable model.

## Results

### Screening and socio-demographic information

**Figure 2.**
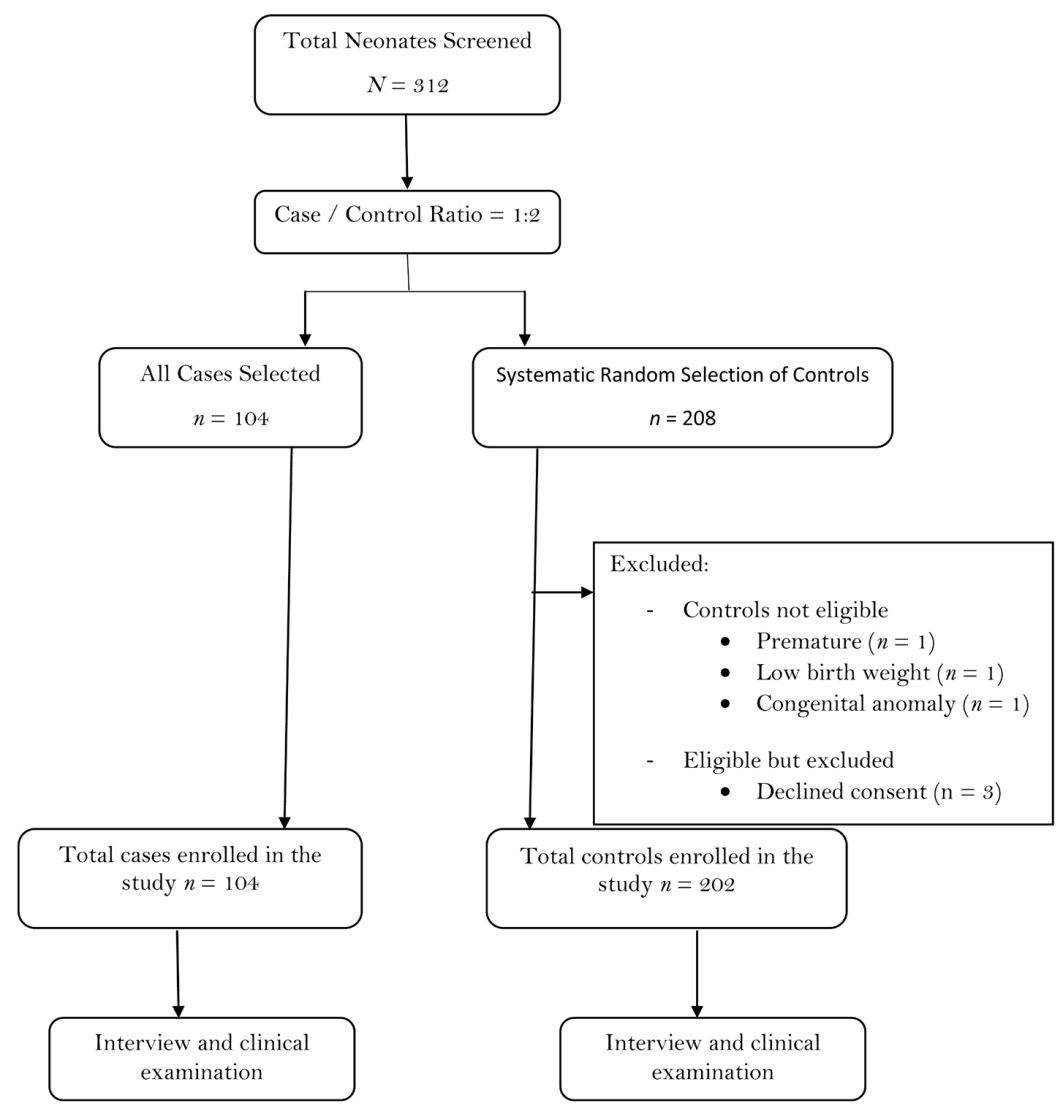
Study flow chart.

A total of 312 participants (104 cases, 208 controls) were recruited into the study but those who consented to participation were 309. Of the 208 potential controls, three declined consent. Additionally, three others did not meet the eligibility criteria and were excluded; leaving 202 eligible controls that participated. A flow diagram illustrating the recruitment and enrollment process is shown in Figure 2.

Descriptive statistics for the demographic variables are indicated in Table 2. Notably, males comprised 55.8% ( *n*=58) of cases and 47.0% ( *n*=95) of controls. The mean neonatal age was 19.7 days; the mean age of cases and controls being 16.5 days (range: 5-28 days) and 21.3 days (range: 3-28 days), respectively. Regarding marital status, 69.2% ( *n*=72) of cases’ mothers were married compared to 81.7% ( *n*=165) of controls’. Only 13.5% ( *n*=14) of the cases’ caregivers had received up to tertiary level of education compared to 22.3% ( *n*=45) of the controls’. 

**Table 2.** Demographic characteristics of the respondents, KHC, Kenya, 2018 (n=306).

Variable	Cases ( *n* = 104)	Controls ( *n* = 202)
Neonate’s sex		
Male	58 (55.77%)	95 (47.03%)
Female	46 (44.23%)	107 (52.97%)
Neonate’s age (days)		
Mean	16.5	21.3
Range	5–28	3–28
Maternal age (years)		
Mean	26.16	27.12
Range	17 – 44	17 – 44
Marital status		
Single	26 (25.00%)	29 (14.36%)
Married	72 (69.23%)	165 (81.68%)
Separated	6 (5.77%)	7 (3.47%)
Divorced	0 (0.00%)	1 (0.50%)
Education Level		
No formal	3 (2.88%)	2 (0.99%)
Primary	36 (34.62%)	62 (30.69%)
Secondary	51 (49.04%)	93 (46.04%)
College/University	14 (13.46%)	45 (22.28%)
Religion		
Protestant	69 (66.35%)	119 (58.91%)
Catholic	23 (22.12%)	61 (30.20%)
Orthodox	10 (9.62%)	21 (10.40%)
Muslim	1 (0.96%)	0 (0.00%)
Pagan	1 (0.96%)	1 (0.50%)

### Cord care practices and umbilical cord hygiene among respondents

A description of the participants’ cord care practices is displayed in Table 3. In this population, majority of mothers reported use of chlorhexidine/surgical spirit (64%, *n*=197). Among cases, slightly over a third (35.6%, *n*=37) had surgical spirit/chlorhexidine applied as compared to about four-fifths (79.2%, *n*=160) of the controls. Of concern, saliva/ash was applied among 10.6% ( *n*=11) of cases compared to 2.5% ( *n*=5) of the controls. In this study setting, about two-thirds (65.7%, n=201) of mothers fastened their babies’ diapers below the umbilical stump. Roughly 30% (29.8%, n=31) of the case respondents revealed that they folded the neonate’s napkin below the cord.

**Table 3.** Cord care practices and cord hygiene among mothers/primary care-givers, KHC, Kenya, 2018 (n=306).

Variable	All mothers (n=306) n (%)	Cases (n=104) n (%)	Controls (n=202) n (%)
Substance application			
Surgical spirit/Chlorhexidine	197 (64.38)	37 (35.58)	160 (79.21)
Topical antibiotic/Silver sulphadiazine	8 (2.61)	2 (1.92)	6 (2.97)
None/Water	85 (27.78)	54 (51.92)	31 (15.35)
Saliva/Ash	16 (5.23)	11 (10.58)	5 (2.48)
Cord exposure			
Below cord	201 (65.69)	31 (29.81)	170 (84.16)
Above cord	105 (34.31)	73 (70.19)	32 (15.84)
Handwashing			
Yes	239 (78.10)	57 (54.81)	182 (90.10)
No	67 (21.90)	47 (45.19)	20 (9.90)
Washing substance			
Water and soap	136 (44.44)	12 (11.54)	124 (61.39)
Water only	103 (33.66)	45 (43.27)	58 (28.71)
None	67 (21.90)	47 (45.19)	20 (9.90)
Rooming-in			
Yes	304 (99.35)	102 (98.08)	202 (100.00)
No	2 (0.65)	2 (1.92)	0 (0.00)
Bathing method			
Sponge-bathing	197 (64.38)	30 (28.85)	167 (82.67)
Immersion in water	109 (35.62)	74 (71.15)	35 (17.33)
Umbilical cord hygiene			
Proper	198 (64.71)	29 (27.88)	169 (83.66)
Improper	108 (35.29)	75 (72.12)	33 (16.34)

Regarding the cleansing substance employed by those who reported handwashing, only 44.4% (n=136) used both water and soap. In particular, whereas 61.4% (n=124) of controls’ mothers stated they used water and soap before cord handling, only 11.6% (n=12) of cases’ mothers did the same. Sponge-bathing was the bathing practice recorded by most (64.4%, n=197) of the participants in the present study. However, only 28.9% (n=30) of the cases were sponge-bathed.

The proportion of mothers/care-givers who had improper hygiene practices was 35.3% (n=108), with unhygienic cord status being disproportionately high in case (72.1%, n=75) than in control mothers (16.3%, n=33), Table 3. 

### Logistic regression analyses

The crude association between cord hygiene and neonatal sepsis is captured in Table 4. Notably, the odds of neonatal sepsis in infants who had improper hygiene was approximately 13 times higher (OR=13.24; 95% CI: [7.5; 23.4]) compared to those with proper hygiene.

**Table 4.** Association between umbilical cord hygiene, neonatal and maternal factors with neonatal sepsis among neonates attending KHC, Kenya, 2018.

Variable	Odds ratio	95% CI	*P*-value
Umbilical cord hygiene			<0.001
Proper	Ref	-	
Improper	13.24	7.50; 23.38	
APGAR score *			
<7	9.47	2.01; 44.70	0.001
>7	Ref		
Invasive procedures *			
Yes	2.84	1.32; 6.10	0.007
No	ref		
Neonate’s sex			
Male	0.70	0.44; 1.13	0.147
Female	ref		
Neonate’s age *	0.89	0.85; 0.93	<0.001
Maternal age (years)			
Mean	0.97	0.93; 1.01	0.159
Level of education			0.187
No formal	2.74	0.44; 16.91	
Primary	1.06	0.62; 1.81	
Secondary	Ref		
College/University	0.57	0.24; 1.13	
Marital status *			
Single	2.05	1.13; 3.73	0.0498
Married	Ref		
Divorced/Separated	1.72	0.56; 5.13	
Mother’s religion			
Protestant	Ref		0.181
Catholic/Orthodox	0.69	0.42; 1.15	
Other	3.45	0.31; 38.74	
Place of delivery			
Home delivery	0.72	0.07; 7.04	0.251
Primary public	1.62	0.94; 2.81	
Public hospital	Ref		
Private hospital	0.85	0.43; 1.67	
Health education			
Received	0.59	0.33; 1.07	0.086
Not received	Ref		
Parity	0.84	0.66; 1.07	0.149
Number of ANC visits			
Zero	Ref		0.525
One	0.5	0.07; 3.65	
Two	0.28	0.04; 1.87	
Three	0.29	0.05; 1.83	
≥Four	0.36	0.06; 2.24	
Immunization			
Immunized	0.51	0.10; 2.56	0.416
Non-immunized	Ref		
Initiation of breastfeeding *			
Within one hour	Ref		0.006
One-six hours	2.85	1.49; 5.43	
More than 6 hours	1.29	0.74; 2.24	
Type of feed *			
Breastmilk only	Ref		<0.001
Formula	1.61	0.26; 9.80	
Mixed/Other	5.26	2.46; 11.25	
Pregnancy-related events *			
Present	2.04	1.24; 3.36	0.005
Absent	Ref		

* Variables eligible for an assessment of their association with the primary exposure ( *P*≤0.05). CI, confidence interval.

Of the variables screened, neonatal factors registering a significant association with neonatal sepsis were: low APGAR score ( *P*=0.001), invasive procedures ( *P*=0.007) and neonate’s age ( *P*<0.001). With respect to maternal factors, marital status ( *P*=0.05), initiation of breastfeeding ( *P*=0.006), type of feed ( *P*<0.001) and pregnancy-related events ( *P*=0.005) were found to be significantly associated with neonatal sepsis ( Table 4). To qualify as potential confounders, these significant factors were further evaluated for an association with the primary exposure as presented in Table 5. Following the assessment, the variables: low APGAR score, invasive procedure, neonate’s age, marital status, type of feed and pregnancy-related events were significantly associated with cord hygiene and thus were offered to the multivariable model to adjust for their potential confounding effect.

**Table 5.** Association between the qualifying covariates and umbilical cord hygiene among neonates at KHC, Kenya, 2018.

Variable	Odds ratio	95% CI	*P*-value
APGAR score ^a^			
>7	Ref	-	0.001
<7	8.91	1.89; 42.02	
Invasive procedures ^b^			
Yes	2.29	1.07; 4.89	0.033
No	ref	-	
Neonate’s age ^c^	0.94	0.91; 0.98	0.001
Marital status ^d^			
Single	2.08	1.15; 3.78	0.047
Married	ref	-	
Divorced/separated	1.62	0.54; 4.83	
Initiation of breastfeeding			
Within one hour	ref	-	0.624
One-six hours	1.05	0.61; 1.81	
More than six hours	1.375	0.72; 2.62	
Type of feed ^e^			
Breastmilk only	ref	-	<0.001
Formula	0.55	0.06; 5.01	
Mixed	4.81	2.25; 10.28	
Pregnancy-related events ^f^			
Yes	1.63	1.00; 2.65	0.046
No	ref	-	

^a,b,c,d,e,f^Variables eligible for inclusion in the multivariable analysis ( *P*≤0.05).

From the multivariable analysis, none of the six factors assessed confounded (resulted in a >30% change in the coefficient for umbilical cord hygiene) the primary association between umbilical cord hygiene and neonatal sepsis ( Table 6), and as such, the OR of 13.34 was used to compute the PAF. The estimated PAF was 66.7% (95% CI: 62.5; 69.0).

**Table 6.** Multivariable analysis for association between umbilical cord hygiene and qualifying covariates with neonatal sepsis among neonates at KHC, Kenya, 2018.

Variable	Odds ratio	95% CI	*P*-value
Umbilical cord hygiene			
Proper	Ref	-	<0.001
Improper	11.02	5.82; 20.87	
APGAR score			
>7	Ref	-	0.328
<7	3.28	0.30; 35.36	
Invasive procedures			
Yes	1.42	0.36; 5.63	0.616
No	Ref	-	
Neonate’s age	0.88	0.84; 0.93	<0.001
Marital status			
Single	1.26	0.57; 2.80	0.836
Married	Ref	-	
Divorced/separated	1.21	0.28; 5.24	
Type of feed			
Breastmilk only	Ref	-	0.009
Formula	4.38	1.60; 11.96	
Mixed	4.26	0.50; 36.44	
Pregnancy-related events ^+^			
Yes	1.56	0.82; 2.99	0.175
No	Ref	-	

None of the assessed factors resulted in a >30% change in the regression coefficient for umbilical cord hygiene.

## Discussion

The study found that in this community, the main cord care procedures involved aspects of substance application, with a majority of caregivers cleansing their hands using water and soap (44%), exposing the cord (66%), sponge-bathing (64%) and practicing rooming-in (99%) of the mother-infant couplet. This is in line with WHO recommendations on satisfactory cord care in high mortality regions: use of select topical antimicrobial agents as alternatives to harmful applications, handwashing, air drying of the umbilical stump, sponge bathing and rooming-in ^16, 20^.

This study found that the most commonly used agents for treatment of the cord were chlorhexidine or surgical spirit (64%). In other studies, similar frequencies in the use of these antimicrobials as the principal cord care application substances have been reported ^41, 45, 46^. However, there was a statistically significant difference between the 36% of cases whose caregivers used surgical spirit/chlorhexidine and the 79% of sepsis-free controls, highlighting the importance of surgical spirit use in prevention of sepsis. Of concern was the significant number of mothers who used non-recommended substances which included water or nothing (air-drying) and ash/saliva. Such unclean substances are a probable nidus of infection as they are likely to be contaminated with bacteria/spores ^24, 45, 47^. In similar settings, variations with respect to the most popularly applied substances have been observed. For instance, in Pumwani Maternity Hospital, Kenya, applying nothing (air drying) was most prevalent at 55%, followed at 25% by surgical spirit, as well as use of saliva and water both at 10% ^40^. Elsewhere, methylated spirit was the main cord care method in Ghana ^48^ and in Nigeria ^41, 46, 49^; while use of brick ash was reportedly highly used in Zambia ^22^. Findings from another study carried out in Benin reported that inappropriate/harmful substances were applied by 81% of caregivers ^50^. In Ethiopia and Nigeria, dry cord care was widely exercised ^51, 52^. The differences might be due to the influence of deeply entrenched cultural norms that supersede adoption of advocated clean cord care applications ^10, 23, 53^.

In this study, about two thirds of mothers tied the babies’ diapers below the umbilical stump. This is in consonance with WHO stipulations that dictate that the diaper should be tied below the cord ^20^. There was a clear distinction among cases and controls, with only 30% of cases and 84% of controls’ mothers reporting to fasten diapers below the cord. A study by Kinanu *et al*. ^40^ found similar results where 54% neonates’ diapers were applied below the cord. The umbilical stump being an acquired wound is a nidus for entry of pathogenic bacteria from the newborn’s excreta ^16, 29, 40^.

About four-fifths (78%) of caregivers mentioned that they washed their hands while changing the diapers (55% of cases and 90% of controls). Another study done in one public hospital in Nairobi, Kenya, supported this finding where 52% of mothers washed their hands under running water and 48% used water in basins ^40^. Comparably, in Parakou, Benin, 73% of mothers expressed that they washed their hands prior to cord care provision ^50^. Further, with regards to the washing substance in the current study, majority of mothers (44%) used water and soap. Mothers who washed with plain water were 37% while those who did not wash their hands at all were 22%. Findings in this study were corroborated by a Nigerian study which documented most of the study population (47%) to have used water and soap in the care of their hands, followed by water only (40%) ^41^. Nonetheless, a study in Karamoja in Uganda, contrasted the finding, reporting that handwashing was not observed by majority (90%) of mothers before change of diaper/napkin leading to their neonates exhibiting signs of infection ^54^. The difference could be ascribed to the Ugandan study area being primarily a semi-arid region of the country compared to the urban setting of this study. While handling the neonate’s cord, it is recommended that handwashing with both water and soap is observed to achieve umbilical cord hygiene ^11, 20^.

Over 99% of the mothers in this study slept in the same room as the baby. The finding from a study in Pumwani Maternity Hospital in Kenya supports this result where 93.3% of mothers were shown to practice rooming-in ^40^. It is recommended that mothers and their newborns should sleep in one room throughout without separation ^16^. It has been cited that rooming-in promotes better coupling of mother and newborn, boosting their skin contact and hence increasing colonization rates of non-pathogenic organisms from the mothers’ normal skin flora to the baby, thereby lowering umbilical cord infection rates ^14, 20, 55^.

To achieve dry cord care and hastened healing, the bathing practice is key. Wiping the baby with a wet cloth was dominant among controls in the present study. Similarly, sponge-bathing has been shown in another study to be the main bathing practice compared to immersion-bathing ^40^. However, majority of cases were immersed in water. In Benin, 93% of mothers tub-bathed babies in water and only 7% wiped them with a wet cloth which was linked to concomitant umbilical cord infection ^50^. The WHO recommends that the first bath should be delayed for at least six hours and umbilical stump should be kept dry until the cord falls off ^20^; the reason being that immersion bathing leads to delay in cord separation and increased susceptibility to sepsis ^56^.

This study results showed that 35.3% of caregivers failed to observe good cord hygiene. In North Benin, a study reported that, as per study’s specifications of cord hygiene, 58.6% of mothers had practiced poor quality care, 31.9% had good quality care, with none of the mothers reaching excellent quality of cord care ^50^. It is noteworthy that, owing to understaffing in most primary care facilities in Kenya, antenatal education on good care may not be adequately provided. Consequently, mothers attending such facilities may resort to improper methods of cord care that may in turn predispose their neonates to sepsis.

The results of the present study demonstrated a statistically significant association between umbilical cord hygiene and neonatal sepsis among infants of the Kahawa Health Centre. Compared to babies whose mothers observed proper cord hygiene, the odds of developing neonatal sepsis among babies of mothers who had improper cord hygiene was roughly 13 times higher (OR=13.24; *P*<0.001) ** and this key association was not confounded by any of the examined factors. According to Bradford Hill criteria, such a strong association has been shown to be less likely due to chance, bias or confounding and might suggest causality ^57^. However, this finding needs to be validated by studies in other settings.

In India, a previous study has elucidated a strong association ( *P*<0.0001) between unhygienic care of the cord and sepsis ^24^. Likewise, a study in Bangladesh showed a relative risk of 1.15 for an association between unclean cord care and neonatal sepsis ^58^. The strength of association is lower than the results of this study perhaps attributable to other stronger predictors of neonatal sepsis in the population. A similar observation was made in Nigeria where unhygienic cord care was strongly associated with neonatal infection ^15^.

With the strong OR, this study yielded a high overall PAF estimate of 67% for umbilical cord hygiene. This implies that in the study's neonatal population, sixty-seven percent of neonatal sepsis cases would have been averted, if good cord hygiene was observed and assuming umbilical cord hygiene was causal. Associations drawn from this study are generalizable to similar low-resource primary care settings.

A few limitations are intrinsic to the present study. Recall of past exposures was likely to be more complete in respondents whose neonates were cases than controls. This could bias the effect estimates away from unity. Moreover, there was likely to be differential reporting of cord care practices between cases’ and controls舗 caregivers, accordingly, biasing the effect estimates away from null. Furthermore, as availability of laboratory diagnostics is limited at KHC, this precluded their inclusion in the case definition and may therefore have affected the definition’s specificity. 

## Conclusion

This study provides evidence that improper cord hygiene is strongly associated with neonatal sepsis among infants presenting in this primary care setting. More importantly, this association was not confounded by any of the covariates measured. The PAF estimate implies that observance of good cord hygiene practices would result in a 67% reduction of sepsis in this neonatal population. Hence, there is a pressing need to spearhead revision of national guidelines with a view to introducing an antenatal cord care package that lays emphasis on the importance of comprehensive cord care practices.

## Data availability

### Underlying data

Harvard Dataverse: Replication Data for: Association between umbilical cord hygiene and neonatal sepsis among neonates presenting to a primary care facility, Kenya: A case-control study. https://doi.org/10.7910/DVN/FSXPR8 ^32^.

This project contains the following underlying data:

- kahawa_hygiene_code.do (.do file code for umbilical cord-neonatal sepsis evaluation).
- kahawa_hygiene_data.tab (study dataset).

### Extended data

Harvard Dataverse: Replication Data for: Association between umbilical cord hygiene and neonatal sepsis among neonates presenting to a primary care facility, Kenya: A case-control study. https://doi.org/10.7910/DVN/FSXPR8 ^32^.

This project contains the following extended data:

- Umbilical hygiene_sepsis questionnaire.pdf (questionnaire used in this study).

Data are available under the terms of the Creative Commons Zero “No rights reserved” data waiver (CC0 1.0 Public domain dedication).
