# Supplementary material for: Association between umbilical cord hygiene and neonatal sepsis among neonates presenting to a primary care facility in Nairobi County, Kenya: a case-control study
Source: F1000Res. 2019 Jul 30;8:920. Originally published 2019 Jun 21. [Version 2] doi: 10.12688/f1000research.19544.2 (PMC6696615; doi:10.12688/f1000research.19544.2)
Supplement: Peer review history

# Peer review: Reviewer response for version 2

**DOI:** 10.5256/f1000research.21993.r51821

**Published:** 2019-08-15

**Authors:** Jamlick Karumbi

**Affiliations:** 1 Ministry of Health, Nairobi, Kenya

My concerns and comments have been adequately addressed.

I confirm that I have read this submission and believe that I have an appropriate level of expertise to confirm that it is of an acceptable scientific standard.

# Peer review: Reviewer response for version 1

**DOI:** 10.5256/f1000research.21426.r50236

**Published:** 2019-07-16

**Authors:** Jamlick Karumbi

**Affiliations:** 1 Ministry of Health, Nairobi, Kenya

This is an important article that clearly describes what may be going wrong in our quest to reduce neonatal mortality in high mortality settings like Kenya. The article does point out that the correct umbilical cord care practices need to be entrenched into routine practices, especially in lower-level facilities. 

 There are a few issues that need to be clarified;

1. In the introduction, the authors state that there are no guidelines for cord care in Kenya, contrary to that there are actually basic pediatric protocols of 2016 here. ****These protocols clearly recommend the use of 4% Chlorhexidine for cord care.
2. The authors used a 1:2 case-control matching, what was the rationale? Why not 1:1? My guess is that this has to do with powering the study enough, if this is the case then it needs to be clearly stated in the methodology.
3. In the results section, Table 3 seems to be entirely described by the text preceding it. I believe the authors can just highlight the key elements in the table and leave the other details in the table.
4. The authors could have enriched the discussion by contextualizing the findings further, for example, commodities are usually a challenge in low-level facilities and also human resources who would have correctly advised the mothers, I was hoping to see this as possible postulations in the discussions and conclusions.

I confirm that I have read this submission and believe that I have an appropriate level of expertise to confirm that it is of an acceptable scientific standard, however I have significant reservations, as outlined above.

# Peer review: Reviewer response for version 1

**DOI:** 10.5256/f1000research.21426.r50237

**Published:** 2019-07-08

**Authors:** Peace Ibo Opara, Balafama Alex-Hart

**Affiliations:** 1 Department of Paediatrics and Child Health, Faculty of Clinical Sciences, University of Port Harcourt, Port Harcourt, Nigeria; 2 Department of Paediatrics, University of Port Harcourt Teaching Hospital, Port Harcourt, Nigeria

This paper describes umbilical cord practices among mothers in a low resource setting and assesses the relationship between umbilical cord hygiene and neonatal sepsis, its impact on the population, as well as the influence of other neonatal and maternal factors on this relationship. The methodology is clearly written and well presented. However, criteria used for cases were purely clinical and do not quite fit into the seven clinical signs identified by WHO especially in developing countries which includes: difficulty feeding, convulsions, movement only when stimulated, respiratory rate >60 per min, severe chest in drawing and axillary temperature >37.5 °C or <35.5 °C ^1^. For example, the authors include an axillary temperature of >37.5 °C but are silent on temperature < 35.5 °C. Other useful signs like cyanosis and grunting are also not included ^2^. It is important to remember that neonatal sepsis shares similar clinical presentations to other common conditions in the neonatal period, thus a combination of clinical and laboratory findings is necessary to provide a correct diagnosis of neonatal sepsis. The limited access to laboratory tests in developing countries ^3^ which perhaps may have been a challenge to the authors should probably be stated if applicable.

 The study describes the statistical methods that were used and the results obtained are clearly stated. Statistical analysis and interpretation of results are adequate. This is a very good paper and is an important addition to the literature on the importance of cord care especially in low resource settings.

We confirm that we have read this submission and believe that we have an appropriate level of expertise to confirm that it is of an acceptable scientific standard.

## References

[1] : Clinical signs that predict severe illness in children under age 2 months: a multicentre study. *Lancet*.2008;371(9607) : 10.1016/S0140-6736(08)60106-3 135-42 10.1016/S0140-6736(08)60106-3 18191685

[2] : What clinical signs best identify severe illness in young infants aged 0-59 days in developing countries? A systematic review. *Arch Dis Child*.2011;96(11) : 10.1136/adc.2010.186049 1052-9 10.1136/adc.2010.186049 21220263PMC3081806

[3] : Neonatal sepsis: progress towards improved outcomes. *J Infect*.2014;68 Suppl 1: 10.1016/j.jinf.2013.09.011 S24-32 10.1016/j.jinf.2013.09.011 24140138
